# Anemia and Kidney Function Decline among the Middle-Aged and Elderly in China: A Population-Based National Longitudinal Study

**DOI:** 10.1155/2020/2303541

**Published:** 2020-10-05

**Authors:** Chao Yang, Qinqin Meng, Huaiyu Wang, Yafeng Wang, Zaiming Su, Lili Liu, Wenwen Liu, Guilan Kong, Luxia Zhang, Yaohui Zhao, Ming-Hui Zhao

**Affiliations:** ^1^Renal Division, Department of Medicine, Peking University First Hospital, Peking University Institute of Nephrology, Beijing, China; ^2^Institute of Social Science Survey, Peking University, Beijing, China; ^3^National Institute of Health Data Science at Peking University, Beijing, China; ^4^Department of Epidemiology and Biostatistics, School of Public Health, Peking University, Beijing, China; ^5^National School of Development, Peking University, Beijing, China; ^6^Peking-Tsinghua Center for Life Sciences, Beijing, China

## Abstract

Chronic kidney disease (CKD) is a public health burden, and anemia is common among patients with CKD. However, less is known regarding the longitudinal association between anemia and deterioration of kidney function among the general population. The China Health and Retirement Longitudinal Study is a nationally representative survey for households with members aged ≥ 45 years. Participants without creatinine and demographic data in 2011 and 2015 were excluded. Anemia was defined according to definitions of the World Health Organization. Rapid decline in kidney function was defined as a ≥16.9% (quartile 3) decline in estimated glomerular filtration rate (eGFR), calculated using the CKD-EPI equation during 2011-2015. Multivariate logistic regression and restricted cubic splines were used to explore their relationship. Altogether, 7210 eligible participants were included in the analysis, with a mean age of 58.6 ± 8.8 years. Rapid decline in kidney function occurred among 1802 (25.0%) participants. Those with kidney function decline were more likely to be older, male, and have anemia, lower eGFRs, hypertension, and cardiovascular disease (*P* < 0.05). Anemia, or hemoglobin, was independently associated with rapid decline in kidney function after adjusting for potential confounding factors (OR = 1.64, 95% CI, 1.32-2.04; OR = 0.90, 95% CI, 0.87-0.94, respectively). Restricted cubic splines showed a nonlinear relationship between hemoglobin and rapid decline in kidney function, especially for men with anemia (*P* < 0.05). In conclusion, anemia is an independent risk factor for progression of kidney function among the middle-aged and elderly population. Attentive management and intervention strategies targeting anemia could be effective to reduce the risk of kidney failure and improve the prognosis of the general population.

## 1. Introduction

Chronic kidney disease (CKD) is a public health burden globally and has affected 8% to 16% of the world's population [1, 2]. Patients with CKD are significantly associated with the risk of end-stage kidney disease (ESKD) and other adverse outcomes including lower cognitive abilities, cardiovascular disease (CVD), and death [3, 4]. It was predicted that CKD would rise from 16th to 5th in the leading causes of early death between 2016 and 2040, which portended serious public health implications [5].

Anemia is one of the most common complications in patients with CKD, especially in those with lower estimated glomerular filtration rate (eGFR) and those with ESKD requiring kidney replacement therapy [6–8]. Data from the National Health and Nutrition Examination Survey (NHANES) in 2007-2008 and 2009-2010 showed that anemia was twice as prevalent in patients with CKD (15.4%) as in the general population (7.6%) in the U.S., and its prevalence increased from 8.4% at CKD stage 1 to 53.4% at CKD stage 5 [9]. Anemia is also associated with increased cardiovascular risks in patients with CKD [10].

Meanwhile, anemia is a risk factor for progression of CKD despite differences in definitions of anemia [11]. The Reduction in End points in Noninsulin-dependent diabetes mellitus with the Angiotensin II Antagonist Losartan (RENAAL) study revealed that even a modest degree of anemia in type 2 diabetic patients with nephropathy was associated with the increased risk for renal outcomes [12]. A more rapid decrease in eGFR was also found between anemic and nonanemic patients with CKD stage 3 (-6.8 mL/min/36 months vs. -1.6 mL/min/36 months) [13]. Furthermore, several intervention studies suggested that early correction of anemia could slow the CKD progression and improve health-related quality of life [14, 15].

However, there is less research and evidence regarding the causative association between anemia and deterioration of kidney function among the general population. Moreover, some observational studies suffer from the bias that anemic patients are often sicker [16]. Therefore, the aim of this study was to investigate the longitudinal association of anemia with kidney function decline among the middle-aged and elderly population in China, using prospective data from the China Health and Retirement Longitudinal Study (CHARLS).

## 2. Materials and Methods

### 2.1. Study Population

The CHARLS is a nationally representative longitudinal survey for households with members aged 45 years and above [17]. The baseline survey was conducted between June 2011 and March 2012, covering 450 villages/urban communities in 28 provinces in China. Since then, the participants have been followed up every two years, using a face-to-face computer-assisted personal interview. Details of the sampling methods have been described previously [17, 18]. Briefly, a four-stage, stratified, cluster sampling method was used to obtain a sample of eligible participants.

**Figure 1:**
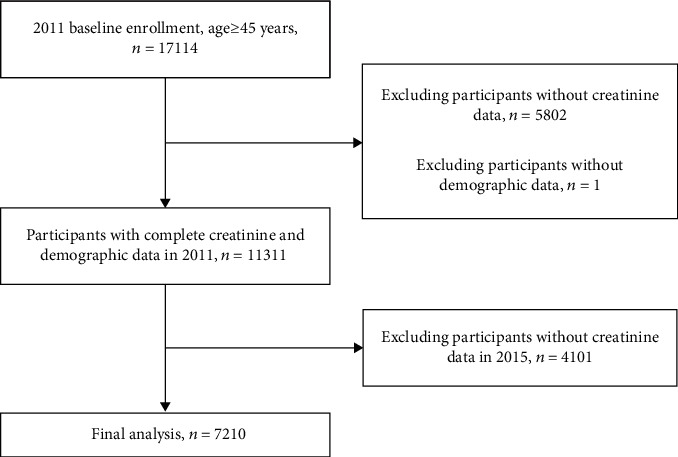
Flow chart of the selection of participants.

Overall, a total of 17114 individual participants aged 45 years and above were included in the 2011 baseline enrollment. Venous blood samples were drawn from 11312 individuals, among which 1 person was further excluded due to missing demographic information. Of the remaining individuals, 7210 individuals donated venous blood again in 2015 (Figure 1). The Biomedical Ethics Review Committee of Peking University approved this study, and all participants gave written informed consent before participation.

### 2.2. Data Collection and Variables

Information on participants' sociodemographic status, lifestyle and health-related behaviors, self-reported comorbidity, social activities, and medications were collected through a structured questionnaire. All study investigators and staff members completed a standardized training program and had a detailed manual of procedures.

Systolic and diastolic blood pressures were measured three times with a 45 s interval using a HEM-7200 electronic monitor (Omron (Dalian) Co., LTD., Dalian, China). The average of three readings was calculated. All blood samples were shipped to Beijing and stored at -70°C. High-sensitivity C-reactive protein (CRP), total cholesterol, high-density lipoprotein (HDL), low-density lipoprotein (LDL), triglycerides, fasting glucose, creatinine, and uric acid were tested [19, 20]. Creatinine levels were measured using a rate-blanked and compensated Jaffe creatinine method. Quality control (QC) samples were used daily during the test. All test results from QC samples were within the target range of two standard deviations (SDs) of mean QC control concentrations [18].

### 2.3. Assessment Criteria

Anemia was defined as hemoglobin levels less than 13 g/dL in men and 12 g/dL in women according to definitions of the World Health Organization [21]. The eGFR was calculated using the Chronic Kidney Disease Epidemiology Collaboration (CKD-EPI) equation [22]. The measurement errors between laboratories were corrected because serum creatinine was measured in different laboratories in 2011 and 2015. Based on results from a total of 150 blood samples that were both tested in these two years, a linear regression model was developed to calibrate creatinine levels in 2011 after excluding outliers: *Y* (calibrated) = 0.7483 × *X* (uncalibrated) + 0.0916, *R*^2^ = 0.88.

Participants were considered as having rapid decline in kidney function if the percentage of decrease in eGFR exceeded quartile 3 (16.9%) during 2011-2015, based on its established use in prior studies [23, 24]. Hypertension was defined as an average systolic blood pressure ≥ 140 mmHg, or an average diastolic blood pressure ≥ 90 mmHg, or self-reported use of antihypertensive medications, or self-reported history of hypertension. Diabetes was defined as fasting plasma glucose ≥ 7.0 mmol/L, by use of hypoglycaemic agents, or any self-reported history of diabetes. Hyperuricaemia was defined as uric acid ≥ 7 mg/dL in men and ≥6 mg/dL in women [25]. Body mass index (BMI) was calculated as weight (kg) divided by height (m) squared and categorized according to the Chinese specific criteria for defining underweight (<18.5 kg/m^2^), normal weight (18.5-24 kg/m^2^), overweight (24-28 kg/m^2^), and obesity (≥28 kg/m^2^). Central obesity was defined as waist circumference ≥ 90 cm in men or ≥80 cm in women.

### 2.4. Statistical Analysis

Characteristics of baseline participants were described according to the level of kidney function. Continuous data were presented as mean (SD) or as median (interquartile range (IQR)) for highly skewed variables, and categorical variables were presented as proportions. To compare differences in variables between groups, the *t*-test, chi-square test, or Mann–Whitney test was used as appropriate.

The association between anemia (or hemoglobin levels) and rapid decline in kidney function was analyzed by multivariate logistic regression, with adjustment for age, sex, residence, education, medical insurance, personal consumption expenditure (PCE), smoking, drinking, BMI, central obesity, CRP, CVD, hypertension, diabetes, hyperuricaemia, and baseline eGFR. Most of these potential confounders have previously been reported to be associated with anemia or kidney function decline, but may not intermediate variables [26–29]. As a result of evaluating collinearity using the variance inflation factor (VIF) and tolerance, all VIF values were less than 10 and all tolerance values were greater than 0.1, indicating no collinearity between the variables included in the model. The adjusted odds ratio (OR) with 95% confidence interval (CI) was present. Restricted cubic splines were used to explore the shape of the association between hemoglobin and rapid decline in kidney function in men and women after adjusting for the above confounding factors. The spline models showed the risks of rapid decline in kidney function per 1 g/dL decrease in hemoglobin, which were represented with ORs and 95% CI. Knots were placed at 11 g/dL and sex-specific tertiles of hemoglobin.

In the sensitivity analysis, participants with baseline eGFR less than 60 mL/min/1.73 m^2^ were excluded to further verify the temporal relationship between anemia and progression of kidney function. All statistical analyses were performed using Stata version 14.0 (StataCorp LP, College Station, TX, USA). Statistical significance was set at two-tailed *P* < 0.05.

## 3. Results

### 3.1. General Characteristics

Characteristics regarding 7210 participants by kidney function are shown in Table 1. The mean age was 58.6 (8.8) years, and 46.2% of participants were male. The proportion of people with anemia was 11.8%. Rapid decline in kidney function occurred among 1802 (25.0%) participants. Those with rapid decline in kidney function were more likely to be older, male, and have high income levels and unhealthy lifestyles (*P* < 0.05). Lower eGFRs, hypertension, CVD, and anemia were more common in participants with rapid decline in kidney function (*P* < 0.05).

### 3.2. Decrease in eGFR

The median of absolute decrease in eGFR and the percentage was 9.8 mL/min/1.73 m^2^ and 9.5%, respectively (Table 2). Compared with participants without anemia, those with anemia had a higher decrease in eGFR (11.0 mL/min/1.73 m^2^ vs. 9.7 mL/min/1.73 m^2^, *P* < 0.05; 11.0% vs. 9.3%, *P* < 0.05). The proportion of rapid decline in kidney function in anemic and nonanemic participants was 31.4% and 24.1%, respectively (*P* < 0.05) (Table 2).

### 3.3. Anemia and Rapid Decline in Kidney Function

**Figure 2:**
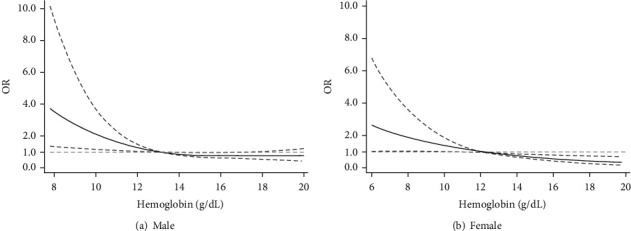
Restricted cubic spline curve for association between hemoglobin and rapid decline in kidney function stratified by gender. Note: the models were adjusted for age, residence, education, medical insurance, personal consumption expenditure, smoking, drinking, body mass index, central obesity, C-reactive protein, cardiovascular disease, hypertension, diabetes, hyperuricaemia, and baseline eGFR. The reference value of hemoglobin is 13 g/dL for men and 12 g/dL for women, respectively. Grey lines represent 95% confidence intervals of OR values. Abbreviations: eGFR: estimated glomerular filtration rate; OR: odds ratio.

After adjusting for potential confounding factors, anemia was independently associated with rapid decline in kidney function (OR = 1.64, 95% CI, 1.32-2.04). The multivariate model based on the hemoglobin levels also found the significant association (OR = 0.90, 95% CI, 0.87-0.94) (Table 3). Similar results were observed among participants with baseline eGFR ≥ 60 mL/min/1.73 m^2^ (Table S1). Restricted cubic splines showed a nonlinear relationship between hemoglobin and rapid decline in kidney function, especially for men with anemia (*P* < 0.05), but the curve for women tended to be linear (Figure 2). Other factors associated with kidney function decline included female, uncontrolled hypertension, elevated CRP, and baseline eGFR (Table S2).

## 4. Discussion and Conclusion

To our knowledge, this is the first study evaluating the longitudinal association of anemia with kidney function decline among the general population, using a nationally representative sample from the CHARLS in China. Our study indicates that anemia is an independent risk factor for the progression of kidney function among the general middle-aged and elderly population, highlighting the importance of specific anemia management in both men and women. The public health implications of our findings can translate into early intervention strategies of anemia among the general population, especially those with risk factors of CKD.

According to data from the China Nutrition and Health Survey in 2010-2012, the prevalence of anemia among urban residents was 9.7% [30]. People aged 45 years and above had a higher prevalence of anemia, which is similar to our results (11.8%). Our study indicated that the overall eGFR decreased by 9.5% during 2011-2015, and the risk of rapid decline in kidney function in anemic patients was 64% higher than that in nonanemic patients. Previous studies regarding the association between anemia and progression of kidney function focused on patients with diabetes or CKD. Meguro et al. found hemoglobin concentration was a significant predictor of kidney function decline in patients with type 2 diabetes mellitus [27]. A recent study based on 439 patients with CKD stage G3 showed decreased kidney function in anemic patients compared to nonanemic participants, as revealed by the faster decrease of eGFR and the shorter time to reach CKD stage G4 or CKD stage G5 [13]. However, evidence from the general population is extremely limited and further research is still needed.

Anemia in CKD is largely due to a deficiency in renal production of adequate amounts of erythropoietin, and suppression of the marrow response to anemia and shortened red cell survival also contribute to anemia in CKD [28, 31]. Conversely, anemia could also contribute to worsening of kidney function, mainly by hypoxia and/or by increased oxidative stress, which is supported by limited data derived from clinical studies [11, 28]. Anemia impairs oxygen delivery to tissues and thus affects organ function including cardiac function. A triad of worsening anemia, worsening CKD, and worsening congestive heart failure creates a vicious cycle referred to as the cardiorenal anemia syndrome [15]. Our study found a nonlinear relationship between hemoglobin and kidney function decline with a little steeper slope for men with anemia, but the curve for women revealed an approximately linear trend. This may be explained by the sex differences in hemoglobin and CKD progression, which is due to factors such as sex hormones, iron utilization, and menstrual blood losses [32, 33]. Although the splines showed that with the decrease of hemoglobin levels, men might have a higher risk of rapid decline in kidney function, it should be noted that the 95% CI of OR values was also getting wider. Previous studies showed that correction of anemia was associated with a reduced loss of residual kidney function [14, 15], but anemia in predialysis CKD patients often goes undetected and untreated, despite the potentially serious and costly consequences [15].

Our study has the advantages of using a large national representative sample and strict quality control measures to ensure data validity and reliability. To our knowledge, this is the first study in the general population that shows the longitudinal association of anemia with progression of kidney function and it may help with causal inference. Moreover, the relationship of hemoglobin and kidney function decline can be intuitively presented by restricted cubic splines. However, there are certain limitations which deserve mention. Firstly, the CHARLS study did not collect the urine samples of participants, which impeded the assessment of CKD progression with slightly increased albuminuria, and markers of kidney damage were measured only once. Secondly, a number of participants were excluded because they did not have demographic or creatinine data, which might lead to potential selection bias. Comparisons of characteristics between the study population and the excluded population are shown in Table S3, indicating that these two groups have similar life styles, but the latter may be dominated by those with higher socioeconomic status. Thirdly, serum creatinine was not measured at the same center in 2011 and 2015, but we built a regression model to correct the measurement bias. Fourthly, the level of random blood glucose was not considered in the definition of diabetes, which might affect the accurate diagnosis of diabetes. Fifthly, since our analysis was based on the general population, we did not adopt the commonly used definition of rapid decline in kidney function as in previous studies [29, 34].

In conclusion, anemia is a risk factor for the progression of kidney function among the middle-aged and elderly general population in China. As China has entered an aging society, the future burden is considerable and needs more concern. Attentive management and interventions targeting anemia could be effective to reduce the risk of kidney failure and improve the prognosis of the general population.

## Figures and Tables

**Table 1:** Baseline characteristics of participants.

Characteristics	Total (*n* = 7210)	Nonrapid decline (*n* = 5408)	Rapid decline (*n* = 1802)	*P* value
Age (years)	58.6 (8.8)	57.9 (8.5)	60.9 (9.2)	<0.001
Male	3333 (46.2)	2373 (43.9)	960 (53.3)	<0.001
Rural residents	6065 (84.1)	4587 (84.8)	1478 (82.0)	0.016
High school education or above	679 (9.4)	518 (9.6)	161 (8.9)	0.426
PCE (yuan), median (IQR)	6258.2 (3871.7, 10463.4)	6137.0 (3862.3, 10283.2)	6703.1 (3965.3, 10957.1)	0.022
Medical insurance	6849 (95.0)	5150 (95.2)	1699 (94.3)	0.154
Current smoker	2120 (29.4)	1543 (28.5)	577 (32.0)	<0.001
Current drinker	2382 (33.0)	1776 (32.8)	606 (33.6)	0.027
Having physical examinations in the past two years	5340 (74.1)	3980 (73.6)	1360 (75.5)	0.100
BMI (kg/m^2^)	23.6 (3.9)	23.7 (3.9)	23.6 (3.8)	0.491
Uric acid (*μ*mol/L)	262.7 (73.5)	255.7 (70.9)	283.6 (77.0)	<0.001
Creatinine (*μ*mol/L)	59.2 (12.2)	57.7 (10.8)	63.7 (14.7)	<0.001
eGFR (mL/min/1.73m^2^)	100.6 (11.1)	101.9 (9.9)	96.6 (13.2)	<0.001
Total cholesterol (mmol/L)	5.0 (1.0)	5.0 (1.0)	5.0 (1.0)	0.397
Triglyceride (mmol/L), median (IQR)	1.2 (0.8, 1.8)	1.2 (0.8, 1.8)	1.2 (0.9, 1.8)	0.732
HDL (mmol/L)	1.3 (0.4)	1.3 (0.4)	1.3 (0.4)	0.031
LDL (mmol/L)	3.0 (0.9)	3.0 (0.9)	3.0 (0.9)	0.601
CRP (mg/L), median (IQR)	1.0 (0.6, 2.1)	1.0 (0.5, 2.0)	1.1 (0.6, 2.3)	<0.001
Hemoglobin (g/dL)	14.4 (2.2)	14.5 (2.2)	14.3 (2.1)	<0.001
Diabetes	1018 (14.1)	758 (14.0)	260 (14.4)	0.571
Hypertension	2577 (35.7)	1840 (34.0)	737 (40.9)	<0.001
CVD	1005 (13.9)	728 (13.5)	277 (15.4)	0.042
Central obesity	1622 (22.5)	1230 (22.7)	392 (21.8)	0.196
Anemia	853 (11.8)	585 (10.8)	268 (14.9)	<0.001

Note: data are *n* (%) or mean (SD), unless stated otherwise. Abbreviations: BMI: body mass index; CRP: C-reactive protein; CVD: cardiovascular disease; eGFR: estimated glomerular filtration rate; HDL: high-density lipoprotein; IQR: interquartile range; LDL: low-density lipoprotein; PCE: personal consumption expenditure.

**Table 2:** The decrease in eGFR of participants from 2011 to 2015.

	Anemia (*n* = 853)	No anemia (*n* = 6357)	Total (*n* = 7210)
Absolute decrease in eGFR (mL/min/1.73m^2^), median (IQR)	11.0 (5.9, 18.5)	9.7 (5.2, 16.5)	9.8 (5.3, 16.9)
Percentage of decrease in eGFR (%), median (IQR)	11.0 (5.9, 19.2)	9.3 (5.1, 16.5)	9.5% (5.2, 16.9)
Rapid decline in kidney function^a^, *n* (%)	268 (31.4%)	1534 (24.1%)	1802 (25.0%)

Note: ^a^rapid decline in kidney function was defined as the percentage of decrease in eGFR exceeded quartile 3 (16.9%) from 2011 to 2015. Abbreviations: eGFR: estimated glomerular filtration rate; IQR: interquartile range.

**Table 3:** Multivariate logistic regression analysis for the association between anemia/hemoglobin and rapid decline in kidney function^a^.

Variable	Adjusted OR^b^	95% CI	*P* value
Anemia			
Male	1.72	1.24–2.39	0.001
Female	1.66	1.24–2.23	0.001
Total	1.64	1.32–2.04	<0.001
Hemoglobin (per 1 g/dL)			
Male	0.92	0.87–0.97	0.003
Female	0.87	0.82–0.92	<0.001
Total	0.90	0.87–0.94	<0.001

Note: ^a^rapid decline in kidney function was defined as the percentage of decrease in eGFR exceeded quartile 3 (16.9%) from 2011 to 2015. ^b^The models were adjusted for age, sex (only in the “total” model), residence, education, medical insurance, personal consumption expenditure, smoking, drinking, body mass index, central obesity, C-reactive protein, cardiovascular disease, hypertension, diabetes, hyperuricaemia, and baseline eGFR. Abbreviations: CI: confidence interval; eGFR: estimated glomerular filtration rate; OR: odds ratio.

## Data Availability

The CHARLS data used to support the findings of this study may be released upon application to the National School of Development, Peking University, who can be contacted at http://charls.pku.edu.cn.

## Supplementary Materials

Supplementary Materials Table S1: the association between anemia/hemoglobin and rapid decline in kidney function (excluding participants with baseline eGFR < 60 mL/min/1.73 m^2^). Table S2: multivariate logistic regression analysis of rapid decline in kidney function. Table S3: comparisons of characteristics between the study population and the excluded population.

## References

[1] Chen T. K., Knicely D. H., Grams M. E. (2019). Chronic kidney disease diagnosis and management: a review. *Journal of the American Medical Association*.

[2] Bikbov B., Purcell C. A., Levey A. S. (2020). Global, regional, and national burden of chronic kidney disease, 1990-2017: a systematic analysis for the Global Burden of Disease Study 2017. *The Lancet*.

[3] Chronic Kidney Disease Prognosis Consortium, Matsushita K., van der Velde M. (2010). Association of estimated glomerular filtration rate and albuminuria with all-cause and cardiovascular mortality in general population cohorts: a collaborative meta-analysis. *Lancet*.

[4] Fujiyoshi A., Miura K., Ohkubo T. (2020). Proteinuria and reduced estimated glomerular filtration rate are independently associated with lower cognitive abilities in apparently healthy community-dwelling elderly men in Japan: a cross-sectional study. *Journal of Epidemiology*.

[5] Foreman K. J., Marquez N., Dolgert A. (2018). Forecasting life expectancy, years of life lost, and all-cause and cause-specific mortality for 250 causes of death: reference and alternative scenarios for 2016-40 for 195 countries and territories. *Lancet*.

[6] McClellan W., Aronoff S. L., Bolton W. K. (2004). The prevalence of anemia in patients with chronic kidney disease. *Current Medical Research and Opinion*.

[7] Astor B. C., Muntner P., Levin A., Eustace J. A., Coresh J. (2002). Association of kidney function with anemia. *Archives of Internal Medicine*.

[8] Nakhoul G., Simon J. F. (2016). Anemia of chronic kidney disease: treat it, but not too aggressively. *Cleveland Clinic Journal of Medicine*.

[9] Stauffer M. E., Fan T. (2014). Prevalence of anemia in chronic kidney disease in the United States. *PLoS One*.

[10] Segall L., Nistor I., Covic A. (2014). Heart failure in patients with chronic kidney disease: a systematic integrative review. *BioMed Research International*.

[11] Rossert J., Froissart M. (2006). Role of anemia in progression of chronic kidney disease. *Seminars in Nephrology*.

[12] Keane W. F., Brenner B. M., de Zeeuw D. (2003). The risk of developing end-stage renal disease in patients with type 2 diabetes and nephropathy: the RENAAL study. *Kidney International*.

[13] NADIR-3 Study Group, Portolés J., Gorriz J. L. (2013). The development of anemia is associated to poor prognosis in NKF/KDOQI stage 3 chronic kidney disease. *BMC Nephrology*.

[14] Gouva C., Nikolopoulos P., Ioannidis J. P., Siamopoulos K. C. (2004). Treating anemia early in renal failure patients slows the decline of renal function: a randomized controlled trial. *Kidney International*.

[15] Dowling T. C. (2007). Prevalence, etiology, and consequences of anemia and clinical and economic benefits of anemia correction in patients with chronic kidney disease: an overview. *American Journal of Health-System Pharmacy*.

[16] Del Vecchio L. (2020). Anaemia epidemiology in the era of “big data”. Are we aware that the revolution is already going on?. *Journal of Nephrology*.

[17] Zhao Y., Hu Y., Smith J. P., Strauss J., Yang G. (2014). Cohort profile: the China Health and Retirement Longitudinal Study (CHARLS). *International Journal of Epidemiology*.

[18] Wang S., Chen R., Liu Q., Shu Z., Zhan S., Li L. (2015). Prevalence, awareness and treatment of chronic kidney disease among middle-aged and elderly: the China Health and Retirement Longitudinal Study. *Nephrology (Carlton, Vic.)*.

[19] Zhao Y., Crimmins E. M., Hu P. (2016). Prevalence, diagnosis, and management of diabetes mellitus among older Chinese: results from the China Health and Retirement Longitudinal Study. *International Journal of Public Health*.

[20] Chen X., Crimmins E., Hu P. P. (2019). Venous blood-based biomarkers in the China Health and Retirement Longitudinal Study: rationale, design, and results from the 2015 wave. *American Journal of Epidemiology*.

[21] World Health Organization (2019). *Iron deficiency anaemia: assessment, prevention, and control*.

[22] Matsushita K., Mahmoodi B. K., Woodward M. (2012). Comparison of risk prediction using the CKD-EPI equation and the MDRD study equation for estimated glomerular filtration rate. *Journal of the American Medical Association*.

[23] Kuwabara M., Bjornstad P., Hisatome I. (2017). Elevated serum uric acid level predicts rapid decline in kidney function. *American Journal of Nephrology*.

[24] Ma X., Zhang C., Su H., Gong X., Kong X. (2018). Increasing body mass index predicts rapid decline in renal function: a 5 year retrospective study. *Hormone and Metabolic Research = Hormon- und Stoffwechselforschung = Hormones et metabolisme*.

[25] Chinese Nephrologist Association (2017). Guidelines for the Diagnosis and Treatment of Hyperuricemia in Renal Diseases in China (2017 edition). *National Medical Journal of China*.

[26] Zhang L., Wang F., Wang L. (2012). Prevalence of chronic kidney disease in China: a cross-sectional survey. *Lancet*.

[27] Meguro S., Tomita M., Kabeya Y. (2012). Factors associated with the decline of kidney function differ among eGFR strata in subjects with type 2 diabetes mellitus. *International Journal of Endocrinology*.

[28] Deicher R., Horl W. H. (2003). Anaemia as a risk factor for the progression of chronic kidney disease. *Current Opinion in Nephrology and Hypertension*.

[29] Young B. A., Katz R., Boulware L. E. (2016). Risk factors for rapid kidney function decline among African Americans: the Jackson Heart Study (JHS). *American Journal of Kidney Diseases*.

[30] Hu Y., Chen J., Li M. (2016). Study on the anemia status of Chinese urban residents in 2010-2012. *Zhonghua Yu Fang Yi Xue Za Zhi[Chinese Journal of Preventive Medicine]*.

[31] Means R. T. (2019). Anemia of renal failure/chronic kidney disease. *Anemia in the Young and Old*.

[32] Rushton D. H., Barth J. H. (2010). What is the evidence for gender differences in ferritin and haemoglobin?. *Critical Reviews in Oncology/Hematology*.

[33] Carrero J. J., Hecking M., Chesnaye N. C., Jager K. J. (2018). Sex and gender disparities in the epidemiology and outcomes of chronic kidney disease. *Nature Reviews. Nephrology*.

[34] Steubl D., Buzkova P., Garimella P. S. (2019). Association of serum uromodulin with ESKD and kidney function decline in the elderly: the Cardiovascular Health Study. *American Journal of Kidney Diseases*.

