# Supplementary material for: Anemia and Kidney Function Decline among the Middle-Aged and Elderly in China: A Population-Based National Longitudinal Study
Source: Biomed Res Int. 2020 Oct 5;2020:2303541. doi: 10.1155/2020/2303541 (PMC7556055; doi:10.1155/2020/2303541)
Supplement: Supplementary Materials — Table S1: the association between anemia/hemoglobin and rapid decline in kidney function (excluding participants with baseline eGFR < 60 mL/min/1.73 m2). Table S2: multivariate logistic regression analysis of rapid decline in kidney function. Table S3: comparisons of characteristics between the study population and the excluded population. [file 2303541.f1.docx]

**Table S1. The association between anemia/hemoglobin and rapid decline in kidney function^a^ (excluding participants with baseline eGFR <60 mL/min/1.73 m^2^)**

| **Variable** | **Adjusted OR^b^** | **95% CI** | ***P* value** |
| --- | --- | --- | --- |
| Anemia |  |  |  |
| Male | 1.83 | 1.31 - 2.54 | <0.001 |
| Female | 1.67 | 1.24 - 2.25 | 0.001 |
| Total | 1.70 | 1.37 - 2.11 | <0.001 |
| Hemoglobin (per 1 g/dL) |  |  |  |
| Male | 0.91 | 0.86 - 0.97 | 0.003 |
| Female | 0.87 | 0.81 - 0.92 | <0.001 |
| Total | 0.90 | 0.86 - 0.93 | <0.001 |

Note: ^a^Rapid decline in kidney function was defined as the percentage of decrease in eGFR exceeded the quartile 3 (16.9%) from 2011 to 2015.

^b^The models were adjusted for age, sex (only in the “total” model), residence, education, medical insurance, personal consumption expenditure, smoking, drinking, body mass index, central obesity, C-reactive protein, cardiovascular disease, hypertension, diabetes, hyperuricaemia, and baseline eGFR.

Abbreviations: CI, confidence interval; eGFR, estimated glomerular filtration rate; OR, odds ratio.

**Table S2. Multivariate logistic regression analysis of rapid decline in kidney function^a^**

| **Variable** | **OR** | **95% CI** | ***P* value** |
| --- | --- | --- | --- |
| Anemia | 1.64 | 1.32 - 2.04 | <0.001 |
| Age (per 10 years) | 1.03 | 0.91 - 1.16 | 0.671 |
| Sex (female) | 0.62 | 0.49 - 0.79 | <0.001 |
| Resident (urban) | 0.91 | 0.73 - 1.14 | 0.422 |
| Education (high school education or above) | 1.04 | 0.79 - 1.36 | 0.802 |
| Medical insurance (Yes) | 0.97 | 0.68 - 1.38 | 0.858 |
| PCE, yuan |  | | |
| Tertile 1 | Reference | | |
| Tertile 2 | 1.12 | 0.93 - 1.35 | 0.227 |
| Tertile 3 | 1.20 | 0.99 - 1.46 | 0.066 |
| CVD | 1.05 | 0.85 - 1.30 | 0.645 |
| Diabetes |  | | |
| No | Reference | | |
| Yes, controlled | 0.93 | 0.74 - 1.18 | 0.555 |
| Yes, uncontrolled | 0.85 | 0.57 - 1.26 | 0.413 |
| Hypertension |  | | |
| No | Reference | | |
| Yes, controlled | 1.16 | 0.93 - 1.45 | 0.183 |
| Yes, uncontrolled | 1.29 | 1.04 - 1.60 | 0.021 |
| Hyperuricaemia | 1.05 | 0.74 - 1.50 | 0.770 |
| CRP, mg/L |  | | |
| Tertile 1 | Reference | | |
| Tertile 2 | 1.20 | 1.00 - 1.45 | 0.056 |
| Tertile 3 | 1.22 | 0.99 - 1.48 | 0.045 |
| BMI, kg/m^2^ |  |  |  |
| Underweight | 1.09 | 0.80 - 1.49 | 0.598 |
| Normal weight | Reference | | |
| Overweight | 1.05 | 0.86 - 1.29 | 0.618 |
| Obesity | 1.32 | 0.90 - 1.93 | 0.163 |
| Central obesity | 0.89 | 0.71 - 1.13 | 0.349 |
| Smoking |  | | |
| Never | Reference | | |
| Current smoker | 1.02 | 0.82 - 1.27 | 0.881 |
| Former smoker | 1.09 | 0.81 - 1.46 | 0.560 |
| Drinking |  | | |
| Never | Reference | | |
| Current drinker | 0.87 | 0.72 - 1.05 | 0.136 |
| Former drinker | 0.80 | 0.60 - 1.05 | 0.109 |
| Baseline eGFR, mL/min/1.73m^2^ | 0.96 | 0.96 - 0.97 | <0.001 |

Note: ^a^Rapid decline in kidney function was defined as the percentage of decrease in eGFR exceeded the quartile 3 (16.9%) from 2011 to 2015.

Abbreviations: BMI, body mass index; CI, confidence interval; CRP, C-reactive protein; CVD, cardiovascular disease; eGFR, estimated glomerular filtration rate; OR, odds ratio; PCE, personal consumption expenditure.

**Table S3. Comparisons of characteristics between the study population and the excluded population**

| **Characteristics** | **Study population (n=7210)** | **Excluded population (n=9904)** |
| --- | --- | --- |
| Age (years) | 58.6 (8.8) | 59.4 (10.6) |
| Male | 3333 (46.2) | 5027 (50.8) |
| Rural residents | 6065 (84.1) | 7265 (73.4) |
| High school education or above | 679 (9.4) | 1502 (15.2) |
| PCE (yuan), median (IQR) | 6258.2 (3871.7, 10463.4) | 7380.4 (4379.0, 13006.5) |
| Medical insurance | 6849 (95.0) | 9124 (92.1) |
| Current smoker | 2120 (29.4) | 2693 (27.2) |
| Current drinker | 2382 (33.0) | 3293 (33.3) |
| Having physical examinations in the past two years | 5340 (74.1) | 7543 (76.2) |

Note: Data are n (%) or mean (SD), unless stated otherwise.

Abbreviations: IQR, inter-quartile range; PCE, personal consumption expenditure.
